# Sun proteins and Dpy19l2 forming LINC-like links are critical for spermiogenesis

**DOI:** 10.1242/bio.016626

**Published:** 2016-05-03

**Authors:** Pierre F. Ray, Charles Coutton, Christophe Arnoult

**Affiliations:** 1 Université Grenoble-Alpes, Grenoble 38000, France; 2 Equipe “Genetics Epigenetics and Therapies of Infertility” Institut Albert Bonniot, INSERM U1209, La Tronche F-38700, France; 3 Laboratoire de Biochimie Génétique et Moléculaire, Institut de Biologie et Pathologie, CHU Grenoble Alpes, Grenoble F-38000, France; 4 Département de Génétique et Procréation, Hôpital Couple Enfant, CHU Grenoble Alpes, Grenoble, F-38000, France

## Abstract

**Summary:** In this response to Pasch et al.’s (2015) discovery that Sun4 is essential for sperm head formation, the authors highlight that like Sun4, Dpy19l2 has a likely LINK-like function and that it also plays a crucial role in spermiogenesis and male infertility.

We have read with a great interest the excellent article by Pasch et al. (2015) which describes the role of Sun4 in sperm head formation and fertility. The authors have used a *Sun4* knockout (*Sun4^−/−^*) mouse model to study the function of Sun4 in spermatogenesis. They observed that homozygous *Sun4^−/−^* males are infertile and present a globozoospermia-like phenotype. Further, they demonstrate that *Sun4* is solely expressed in spermatids and that it localizes to the posterior nuclear envelope, and is excluded from the nuclear membrane facing the acrosome. Sun proteins have been described to link with KASH domain proteins to form LINC complexes that serve to connect the nucleus to the cytoskeleton or to specific organelles. Theses bridges are critical to many cellular processes ranging from cell migration and polarity to chromosomal movement. Until recently, these evolutionarily conserved proteins were the only proteins described as serving this structural function. The authors revealed that Sun4 deficiency leads to the mislocalization of other LINC components and interferes with the formation of the microtubule manchette and prevents the proper positioning of the acrosome and the adequate elongation of the sperm nucleus.

We greatly appreciated Pasch et al.'s manuscript which brings some very interesting and important data regarding acrosome formation and sperm head elongation. We however feel that some additional elements could enrich the discussion. Pasch et al. (2015) quote Frohnert et al. (2011) who described that Sun5 is localized on the spermatids' nuclear membrane facing the acrosome, in opposition to what the authors describe for Sun4 (Frohnert et al., 2011). We would like to highlight that we have published some contradictory data demonstrating that, in fact, Sun5 has a localization very similar to Sun4 and is excluded from the anterior part of the nucleus where the acrosome is attached, indicating that Sun5 does not interact with the acrosome (Yassine et al., 2015). This ‘new’ colocalization of Sun4 and Sun5 thus suggests that the two proteins could interact functionally.

Moreover, Pasch and colleagues mention that the absence of Sun4 leads to the formation of globozoospermia-like sperm. Globozoospermia is characterized by an absence of the acrosome and the formation of a perfectly spherical sperm head, a phenotype not observed in the case of *Sun4^−/−^* sperm as showed in Fig. 5D; the authors therefore adeptly describe their phenotype as globozoospermia-like sperm phenotype (Pasch et al., 2015). We previously showed that the absence of DPY19L2 induces a pure globozoospermia phenotype with perfectly round acrosomeless sperm (Harbuz et al., 2011; Coutton et al., 2012, 2013, 2015). Furthermore, we showed that Dpy19l2 is a transmembrane protein which localizes on the spermatid's inner nuclear membrane on the anterior part of the nucleus facing the acrosome (Pierre et al., 2012). We also demonstrated that in *Dpy19l2^−/−^* mice, the acrosome is not retained on the nuclear membrane and is then eliminated with the cytoplasm. We concluded that Dpy19l2 serves to anchor the acrosome to the nucleus (Pierre et al., 2012). Others have indicated that Dpy19l1, a paralog of Dpy19l12, is necessary for proper radial migration of glutamatergic neurons, thus also displaying a LINC-like function (Watanabe et al., 2011). We can therefore conclude that Dpy19-like proteins may be part of a new family of LINC-like proteins. Pasch et al. conclude by indicating that their study provides evidence for a critical role of LINC complexes in mammalian sperm head formation. We do not dispute this conclusion but would like to add that Dpy19l2 and its yet-unknown partner(s) also play a critical role in spermiogenesis and male infertility, likely by fulfilling a LINC-like function.
